# Predictive role of Glutathione S-transferases (GSTs) on the prognosis of osteosarcoma patients treated with chemotherapy

**DOI:** 10.12669/pjms.295.3870

**Published:** 2013

**Authors:** Jia-wen Teng, Zeng-min Yang, Jie Li, Bo Xu

**Affiliations:** 1 Jia-wen Teng, Orthopedics Department, The Affiliated Hospital of Shandong Traditional Chinese Medicine University,Jinan, China.; 2 Zeng-min Yang,Orthopedics Department, Nanjing Traditional Chinese and Western Medicine Hospital,Nanjing, China.; 3 Jie Li, Orthopedics Department, The Affiliated Hospital of Shandong Traditional Chinese Medicine University,Jinan, China.; 4 Bo Xu, Orthopedics Department, The Affiliated Hospital of Shandong Traditional Chinese Medicine University,Jinan, China.

**Keywords:** GSTs, Osteosarcoma, Chemotherapy, Polymorphisms

## Abstract

***Objective:*** We conducted a comprehensive study to investigate the role of GSTM1, GSTTI and GSTP1 genetic variation involved in transport pathways in response to chemotherapy and clinical outcome of osteosarcoma.

***Methods:*** A total of 146 patients were included in our study between January 2008 and December 2009. All the patients were followed up to death or January 2012. Genotyping of GSTM1, GSTT1 and GSTP1 was conducted in a 384-well plate format on the Sequenom MassARRAY platform.

***Results:*** Sixty seven patients (45.9%) died during the follow-up period. The median age of patients was 14.2 years and ranged from 9.3 to 38.7 years. The median follow-up time was 29.6 months (range 5 to 60 months). Individuals with GSTP1 G/G genotype tended to live shorter than A/A genotype, and we found a significantly higher risk of death from osteosarcoma (adjusted HR=2.73, 95% CI=1.05-7.45). Individuals with the GSTP GG genotype were more likely to have a poor response to chemotherapy, with an OR of 2.73 (95%CI, 1.07-7.81). However, we did not find association of polymorphisms in GSTM1 and GSTT1 with response to chemotherapy and prognosis of osteosarcoma.

***Conclusion:*** Our study provides information for prediction of treatment outcome in clinical oncology. Due to the limited number of samples, the results of our study need to be confirmed by large sample size studies.

## INTRODUCTION

Osteosarcoma is the most common malignant tumor of bones and one of the leading cause of death from cancer in children and adolescents.^1^ Standard treatment of osteosarcoma involves neoadjuvant therapy before surgical resection of the primary tumor, and followed by chemotherapy after operation.^2^ The main chemotherapeutic regimen for osteosarcoma includes methotrexate, cisplatin, cyclophospamide, vincristine or adriamycin. Despite this, about 30% of these osteosarcoma patients, which underwent standard protocol of treatment show recurrence or metastasis during five years period.^1^

Individualized chemotherapy administered taking into account biomarkers’ expression may improve the response to chemotherapy and clinical outcome of patients. Therefore, better understanding of the role of pharmacogenetics could help establishing an individualized chemotherapy and patients may benefit more from chemotherapy to prolong their life, as the genes which influence the clinical response to chemotherapeutics, control drug absorption, distribution, metabolism and excretion.

Glutathione S-transferases (GSTs) are a family of cytosolic enzymes, and they play an important role in the detoxification of various exogenous and endogenous reactive species.^3^^,^^4^ GSTM1, GSTT1 and GSTP1 have been suggested to be involved in detoxification of polycyclic aromatic hydrocarbons (PAHs) and benzo(a)pyrene, which detoxify carcinogens and reactive oxygen species.^5^ Individuals who have homozygous deletions for GSTM1, GSTT1 and GSTP1 gene have reduced enzyme function. Lack of these enzymes potentially increase cancer susceptibility due to a decreased ability to detoxify carcinogens such as benzo[α]pyrene-7,8-diol epoxide, the activated form of benzo[α]pyrene. The missense substitution Ile105Val results from an A3G base substitution at nucleotide 313. The Val105 form of the GSTP1 enzyme is 2–3 times less stable than the canonical Ile105 form and may be associated with a higher level of DNA adducts.^6^^,^^7^

Numbers of published studies have focused on GSTM1, GSTTI and GSTP1 genetic variation with respect to various cancers, but the role of GSTM1, GSTTI and GSTP1 genetic variation in osteosarcoma survival only discussed in a study conducted in China.^8^ Therefore, we conducted a comprehensive study to investigate the role of GSTM1, GSTTI and GSTP1 genetic variation involved in transport pathways in response to chemotherapy and clinical outcome of osteosarcoma.

## METHODS

Total 146 consecutive patients diagnosed with osteosarcoma at Department of Pediatric Orthopedics of Shanghai Children’s Medical Center of Shanghai Jiaotong University between January 2008 and December 2009 were included in our study. Clinical data recorded at study entry included age at diagnosis. All the blood samples were provided by all patients, and written informed consents were gained from patients or their relatives. Our study was approved by the ethics committee of Shanghai Children’s Medical Center of Shanghai Jiaotong University.

The chemotherapy before surgery included intravenous 25-30 mg/m^2 ^adriamycin for three courses at day one, 14 mg/m^2 ^methotrexate for four courses at day one, and intra-arterial 35 mg/m^2 ^cisplatin for three courses at the third day. The adjuvant chemotherapy after surgery included 10 g/m^2^ methotrexate at day one, and alternate cycles of 0.45 mg/m^2^ cisplatin or actinomycin D and 1.5 mg/m^2^ vincristine at day one. The treatments were suspended if patients showed disease progression or serious toxicity. If patients showed three grades of non-hematology toxicity, four grades of hematology toxicity, febrile neutropenia or infection, the dosage of chemotherapeutic drug was reduced by 25% at the next cycle.

The treatment response was determined by the extent of tumor necrosis. Patients with less than 90% necrosis were classified as poor responders and those with 90% necrosis or more, as good responders.^8^ Our primary end point was overall survival (OS) calculated as the time from diagnosis until death from any cause or last known date alive. All the patients were followed up to death or January 2012.

***Genotyping*** ***: ***5 ml venous blood was drawn from all patients, and was kept in -20℃. Genomic DNA was extracted using the TIANamp blood DNA kit (Tiangen Biotech, Beijing, China) with centrifuging for 3 minutes at 13.400 x g (12.000 rpm). Genotyping of GSTM1, GSTT1 and GSTP1 polymorphisms was performed in a 384-well plate format on the Sequenom MassARRAY platform (Sequenom, San Diego, USA). Primers for polymerase chain reaction amplification and single base extension assays were designed using Sequenom Assay Design 3.1 software (Sequenom®) according to the manufacturer’s instructions. PCR was carried out in a reaction volume of 20μl, containing 50 ng of genomic DNA, 200μM dNTP, 2.5 units of Taq DNA polymerase (Promega Corporation, Madison, WI, USA) and 200μM of primers. The conditions of the PCR were as follows: 94˚C for 2 min, 35 cycles of 94˚C for 30 sec, an annealing temperature reduced to 64˚C for 30 sec and 72˚C for 1 minute. The PCR products were analyzed using electrophoresis on 1.0% agarose gel.

***Statistical analysis: ***All analyses were performed using the Statistical Package for the Social Sciences (SPSS) software 13.0 for windows. Correlation between polymorphisms in GSTM1, GSTTI and GSTP1 response to chemotherapy were assessed using odds ratios (95% confident interval) with logistic regression analysis by comparing genotype frequencies in good and poor responders. The association between variants of GSTM1, GSTTI and GSTP1 genotypes and OS was assessed by Cox proportional hazards model with hazard ratios (HR) and their confidence intervals (CI). OS curves were plotted using the Kaplan-Meier method. The All P values were two-tailed, and difference was considered statistically significant when a value of P<0.05. 

## RESULTS

***Study population: ***The main clinical and pathological characteristics of 146 patients are presented in Table-I. Sixty seven patients (45.9%) died during the follow-up period. The median age of patients was 14.2 years and ranged from 9.3 to 38.7 years, and 90 (61.6%) of the patients were males. At the time of diagnosis, 27 (18.4%) of the patients already presented with metastasis, while 32 (21.9%) patients developed metastasis during follow-up. The percentage of good responders to therapy was 52.7% (77 patients), and poor responders were 47.3% (69 patients). The median follow-up time was 29.6 months (range 5 to 60 months).

**Table-I:** Clinical and pathological characteristics of subjects

*Age at diagnosis, y*	*Patients, N*	*%*
Total number of patients	146	
Median (range)	14.2 (9.3-38.7)	
Sex		
Male	90	61.6
Female	56	38.4
Tumor location		
Femur	71	48.7
Tibia/fibula	49	33.6
	15	10.3
Central	11	7.4
Histological response		
Good	77	52.7
Poor	69	47.3
Metastasis at diagnosis		
No	119	81.6
Yes	27	18.4

Our analysis detected a significant effect of GSTP1 polymorphisms on responses to chemotherapy (P < 0.05, Table-II). Individuals with the GSTP GG genotype were more likely to have a poor response to chemotherapy, with an OR of 2.73 (95%CI, 1.07-7.81). However, we did not find any association of GSTT1 and GSTM1 with responses to chemotherapy.

**Table-II:** Correlation of GSTs polymorphisms with tumor response

*Genotype*		*Patients*	*Tumor response*				*OR(95% CI)*	*P value*
			*Poor*	*%*	*Good*	*%*		
GSTP1 313A>G	AA	57	22	32.1	35	45.3	-	-
	AG	57	28	40.5	29	37.5	1.74(0.78-3.76)	0.19
	GG	32	19	27.4	13	17.2	2.73(1.07-7.81)	<0.05
GSTT1	Present	65	29	41.8	36	46.8	-	-
	Null	81	40	58.2	41	53.2	1.33(0.63-2.76)	0.53
GSTM1	Present	87	40	57.8	47	61.2	-	-
	Null	59	29	42.2	30	38.8	1.26(0.67-2.48)	0.61

**Fig.1:**
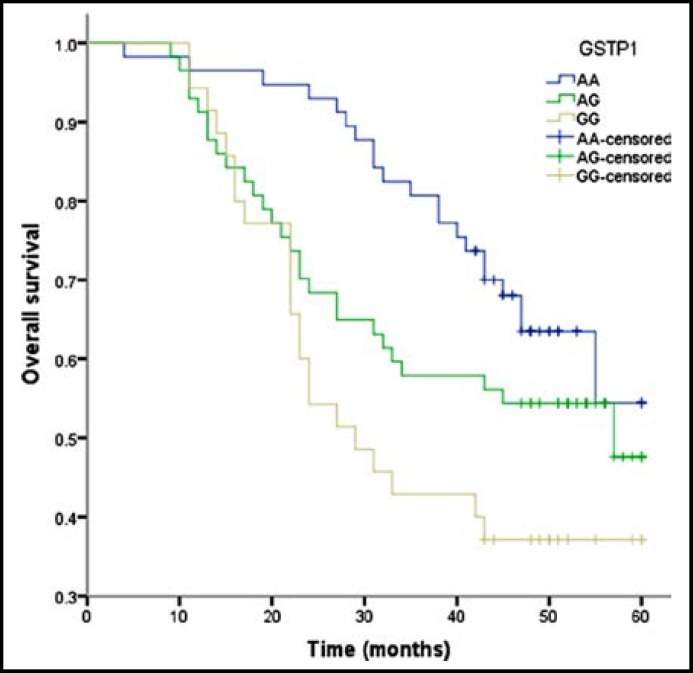
Kaplan-Meier curves of GSTP1 polymorphisms for overall survival of osteosarcoma patients

The relationship of GSTM1, GSTT1 and GSTP1 gene polymorphisms with prognosis of osteosarcoma is shown in Table-III. Polymorphisms in null GSTM1 and GSTT1 had a higher event free survival rate than non-null genotype, whereas no significant association was found between the two genotypes and prognosis of osteosarcoma. Individuals with GSTP1 G/G genotype tended to live shorter than A/A genotype (Fig.1) and we found a significantly higher risk of death from osteosarcoma (adjusted HR=2.73, 95% CI=1.05-7.45).

**Table-III:** Correlation of GSTs polymorphisms with survival of osteosarcoma

*Genotype*		*Cases,*	*%*	*Events,*	*%*	*Survival rate, %*	*Survival, HR (95%)* ^1^	
		*N*		*N*			*Adjusted*	*P value*
GSTP1								
	AA	86	58.8	29	43.5	34.0	-	-
	AG	74	50.5	22	32.7	29.7	1.61(072-3.69)	0.18
	GG	41	27.7	16	23.8	39.4	2.73(1.05-7.45)	<0.05
GSTT1	Present	88	60.3	30	44.6	34.0	-	-
	Null	112	76.7	37	55.4	33.1	1.43(0.72-3.04)	0.31
GSTM1	Present	120	82.1	40	59.7	33.4	-	-
	Null	80	54.9	27	40.3	33.7	1.52(0.73-3.04)	0.24

1. Adjusted for sex, age, subtype, location, metastasis and necrosis

## DISCUSSION

The results of the present study support the pharmacogenetic role of GSTP1 IIe105Val polymorphism in patients with osteosarcoma treated with adjuvant chemotherapy. The GSTP1 GG genotype was associated with poor OS, indicating the potential role of GSTP1 IIe105Val polymorphism in the individualized tailoring chemotherapy for osteosarcoma.

Increasing evidence has suggested an important role for GSTP1 in determining interindividual variations in therapeutic response. It is well known that GSTs is a family of multifunctional enzymes, and this type of enzyme could detoxify a variety of electrophilic compounds. Recent studies indicated that genetic polymorphism in GSTP1 and GSTM1 genes play a role in the efficacy of detoxifying cytotoxins generated by chemotherapeutics, including cisplatin and platinum agents, such as adriamycin, methotrexate, cisplatin and vincristine.^9^^-^^13^ Our study indicated that GSTP1 Ile105Val polymorphism may influence the chemotherapy efficacy in patients with osteosarcoma, and individuals with GSTP1 GG genotype had poor response to chemotherapy and a shorter survival time. Together with existing data on the prevalent expression of GSTP1 in cancer cells, our study findings are in line with previous vitro experiment, indicating that the human 105 Val variant of the GSTP1 enzyme was significantly more active against cisplatin than the enzyme containing the IIe residue.^14^^,^^15^ Our results are in line with previous clinical studies.

Previous clinical studies have confirmed that GSTP1 IIe105Val polymorphisms is associated with cisplatin chemotherapy response.^16^^,^^17^ However, some other studies have reported negative associations between GSTP IIe105Val polymorphism and survival of cancer,^9^^,^^18^^,^^19^ or no association between them.^20^^,^^21^ Two studies conducted in China indicated the GSTP1 polymorphism is correlated with osteosarcoma patients receiving chemotherapy.^9^^,^^11^ Zhang et al reported that a study conducted in China with 159 patients, it indicated that GSTP1 GG genotype tended to liver shorter than the AA genotype.^12^ Another study also conducted in China reported that individuals carrying GSTP1 GG genotype had a lower risk of death from osteosarcoma, with the adjusted HR of 0.32.^9^ Two studies conducted in Brazil and Italy found GSTM1 and GSTT1 polymorphisms may have a role in treatment response and progression of osteosarcoma, but no association between GSTP1 and progression of osteosarcoma.^23^^,^^24^ These contradictions of these results may be caused by differences in the chemical structures or reaction kinetics of these chemotherapy drugs, such as cisplatin and platinum agents. Further large sample studies are greatly warranted to confirm their association.

There are two limitations in our study. Firstly, since our study was conducted in one places, selection bias is inevitable and the results may have limitations for other populations. Secondly, the sample size in our study is relatively small, which would reduce the statistical power to find the differences between groups. Therefore, further large sample multicentre studies are greatly needed.

We further analyzed the role of GSTT1 and GSTM1 polymorphisms in the response to chemotherapy and survival of patients with osteosarcoma. We found the deletion genotypes of GSTT1 and GSTM1 are association with a poor response to chemotherapy and short survival, but no statistically significant association was found between them. Previous studies have indicated that variants of GSTT1 and GSTM1 did not associate with prognosis of osteosarcoma,^9^^,^^11^ which is in line with the results of our study.

## CONCLUSION

Our data showed the polymorphism of GSTP1 appears to be independent prognostic factors in osteosarcoma patients receiving chemotherapy, and GSTM1 and GSTT1 polymorphisms have no statistically significant association with osteosarcoma patients. Our study provides information for prediction of treatment outcome in clinical oncology. Due to the limited number of samples, the results of our study need to be confirmed by large sample size studies.
